# Super‐size me: adipose tissue‐equivalent additions for anthropomorphic phantoms

**DOI:** 10.1120/jacmp.v15i6.5007

**Published:** 2014-11-08

**Authors:** Ryan F. Fisher, David E. Hintenlang

**Affiliations:** ^1^ Imaging Institute, Cleveland Clinic Cleveland OH; ^2^ J. Crayton Pruitt Family Department of Biomedical Engineering University of Florida Gainesville FL USA

**Keywords:** anthropomorphic phantom, tissue‐equivalent material, dosimetry

## Abstract

Physical anthropomorphic phantoms have been utilized for a variety of dosimetric studies across a range of procedures utilizing diagnostic imaging equipment. Unfortunately, these phantoms are often limited to a single reference size, which often may not be representative of the patient population at large. This work set out to develop an adipose tissue‐equivalent substitute material that could be used to create low cost physical additions for existing anthropomorphic phantoms. Using commercially available products, a methodology was developed to accomplish this, and an addition was built to create a 90th percentile by weight phantom from an existing 50th percentile model. This methodology can easily be used to expand the utility of existing anthropomorphic phantoms in order to better represent patients of various body morphologies, and investigate the effects of patient size in diagnostic procedures.

PACS numbers: 87.53.Bn, 87.57.qh

## I. INTRODUCTION

Anthropomorphic phantoms have long been in use for radiation dosimetry studies. Commercially available offerings, such as the RANDO (The Phantom Laboratory, Salem, NY) and ATOM (Computerized Imaging Reference Systems, Inc. Norfolk, VA) phantoms, as well as custom phantoms,^(1)^ have seen use with optically stimulated luminescence (OSL), semiconductor, and fiber optic coupled dosimeters in order to measure skin and organ doses from a range of radiographic and therapy procedures.^2^, ^3^, ^4^, ^5^, ^6^ In order to make physical dose measurements more applicable to the range of clinical patient sizes, it would be beneficial to have phantoms of various body mass indexes (BMI). Unfortunately, commercially available phantoms are generally offered in a limited range of patient sizes. The RANDO phantoms are only available in a single reference male or female model. The ATOM phantom series comprises six phantoms, including a newborn, several pediatric sizes, and adult male and adult female models. Though multiple age options are available, each of these also comes only as a single reference size. Additionally, the high cost of these phantoms makes owning a range of phantom sizes cost‐prohibitive. Custom‐built phantoms, such as those produced at the University of Florida,^(1)^ can be manufactured based on any desired patient size, but the amount of time and labor required for their construction makes it impractical to build multiple phantoms of various body compositions.

The goal of this work was to develop a low cost adipose tissue‐equivalent substitute material for the purpose of fabricating physical additions for existing anthropomorphic phantoms. These additions would allow a single base phantom to be utilized to represent a range of possible patient body mass indexes for the purposes of radiation dose measurements, extending the utility of the phantoms and perhaps bringing their dimensions more in line with those of the general population.

Patient size can have a drastic effect on image quality and absorbed dose for radiological exams. For example, modern computed tomography (CT) scanners make use of automatic tube current modulation systems that vary tube output in response to patient attenuation characteristics. These systems typically involve a user setting a desired image quality level setting in the exam protocol, which the scanner then attempts to match by varying tube output throughout the scan. Using a single image quality level setting across a large range of patient sizes could potentially lead to excessive doses in larger patients. Conversely, lowering the image quality level setting too much for larger patients in order to keep dose levels consistent with smaller patients could lead to unacceptable image quality. The availability of anthropomorphic phantoms representing a range of patient sizes could allow for better optimization of CT protocols by allowing for image quality and dose measurements to be made in order to determine the impact of patient size on exam protocols.

While a custom anthropomorphic phantom was used as the foundation in this work, the methodology described for the construction of the phantom addition is applicable to any of the commercially available, segmented, anthropomorphic phantoms previously described.

## II. MATERIALS AND METHODS

### A. Adipose tissue‐equivalent material development

In order to build an addition for an existing anthropomorphic phantom, an adipose tissue‐equivalent substitute (ATES) had to be developed that would match the X‐ray attenuation characteristics of subcutaneous adipose tissue. For the purposes of this particular study, it was desired to match the attenuation characteristics of adipose for a diagnostic computed tomography (CT) beam. A commercially available, two‐part urethane rubber material, PMC 121/30 Dry, manufactured by Smooth‐On (Easton, PA) was used as a base for the development of the ATES. This material comes as two viscous liquid parts that are mixed in a 1:1 ratio by volume, which cures in a matter of hours into a solid, rubber material.

**Figure 1:**
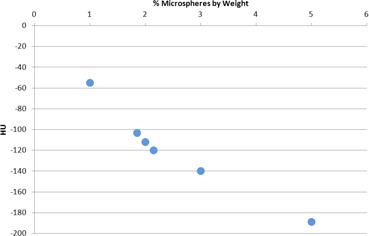
HU value of various samples of ATES material as a function of microsphere concentration in the urethane base material.

A 1999 *Radiology* paper by Yoshizumi et al.^(7)^ was used as a reference for determining an appropriate Hounsfield unit (HU) target for the material. According to their results, based on CT scans of 120 patients over a range of ages and body compositions, the average HU value of subcutaneous abdominal fat was found to be −93±25. The PMC 121/30 Dry material by itself has a higher HU value than this adipose tissue target, so additives were used to modify the X‐ray attenuation properties. Material samples with various concentrations of low density phenolic microspheres (System Three, Auburn, WA) were created and subsequently scanned in a Siemens CT scanner at 120 kV (Siemens Medical Solutions, Malvern, PA). The samples used were slabs measuring approximately 15cm×15cm×4cm, which were scanned while sandwiched between two CTDI head phantoms resting on the examination table. The HU values of the various samples were recorded, and further samples were created based on this initial data. Using this iterative method, a concentration of 2% by weight phenolic microspheres mixed with the PMC‐121/30 Dry material was selected for the final recipe for the ATES, producing a HU value of approximately −100. A graph showing the results of the iterative sample testing is shown in Fig. 1, with each point on the graph representing a material sample of a given concentration of microspheres and its corresponding measured HU value after scanning. The density of the final ATES material was measured to be $0.88\;g/{\text{cm}}^{3}$, which matches well with published values for subcutaneous fat $\left(0.9\;g/{\text{cm}}^{3}\right)$.

### B. Phantom construction

#### B.1 90th percentile hybrid‐computational phantom

**Figure 2:**
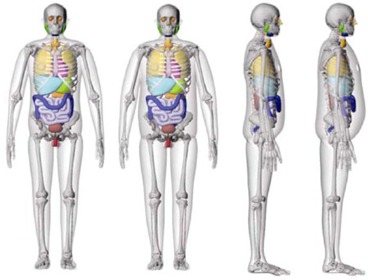
50th and 90th percentile, by weight, hybrid‐computational male phantoms. [Reprinted and adapted with permission from Johnson P. Hybrid Patient‐Dependent Phantoms Covering Statistical Distributions of Body Morphometry in the U.S. Adult and Pediatric Population. PIEEE. 2009;97:2060–2075. p. 2068, Figure 2).]

Once a suitable ATES material had been developed, methods for utilizing it to produce anthropomorphic phantom additions were explored. The base phantom utilized for this work was a custom built, 50th percentile by height and weight adult male phantom, similar to that described by Winslow et al.^(1)^ This phantom was constructed in 5 mm slices, based on data from a hybrid‐computational phantom series created at the University of Florida.^(8)^ This computational phantom series also included a 50th percentile by height, but 90th percentile by weight, male, which served as the model for the ATES addition. This 90th percentile computational phantom was matched to target parameters of standing height, sitting height, and total body mass, as well as physical dimensions such as waist, arm, buttocks, and thigh circumference, as defined by the National Center for Health Statistics databases. The base phantom has a standing height of 5 feet 8 inches and the 90th percentile by weight phantom has a total weight of 214 lbs (BMI 32.5), as compared to the 172 lbs of the 50th percentile phantom (BMI 26.1). Both the 50th and 90th percentile hybrid‐computational phantoms are shown in Fig. 2.

The 50th percentile anthropomorphic phantom was constructed in 5 mm slices which were glued together into 18 sections of variable thickness (ranging from 15–85 mm) in order to allow for dosimeter placement at specific organ locations. The ATES addition was designed and built in sections matching the thickness of the glued sections of the 50th percentile phantom. The decision to build the addition in larger sections was made in order to make the fabrication process more efficient. Due to the larger sections, the resulting 90th percentile phantom has a lower z‐axis resolution compared to the 50th percentile phantom.

#### B.2 Phantom fabrication process

**Figure 3:**
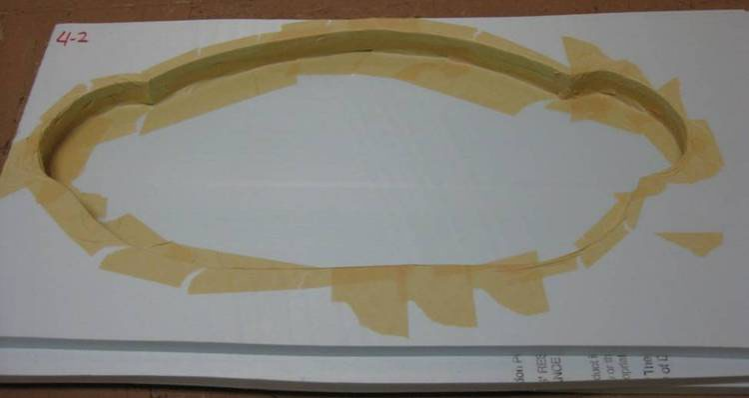
Completed 90th percentile phantom mold. Multiple 1 cm sheets of foam were milled and held together and sealed with masking tape.

In order to build the ATES phantom addition, an automated engraving system (Vision Engraving and Routing Systems, Phoenix, AZ) was used to mill molds out of 1 cm thick extruded polystyrene foam insulation board commonly used for housing insulation. As such, the z‐axis resolution for the process was limited to the thickness of the foam board used. For each 1 cm thick section along the z‐axis of the 90th percentile computational phantom, a bitmap image of the outer body contour was created. In cases where this contour varied throughout the 1 cm thickness, the largest outer body contour of the section was used. These bitmap images were loaded into the engraver software and used to cut the corresponding shapes for each slice comprising the mold. For the larger sections of the phantom, multiple pieces of foam were milled and then stacked to match the z‐axis thickness of the 50th percentile physical phantom section. These stacked sections of foam were sealed with masking tape to produce a mold for the urethane ATES material. A completed body section mold is shown in Fig. 3.

**Figure 4:**
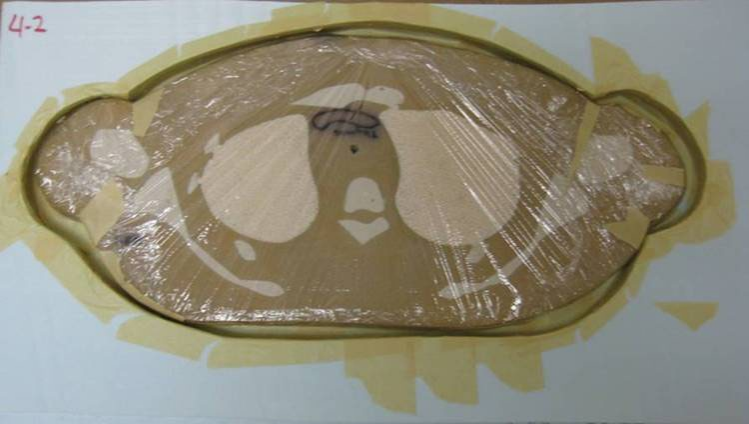
50th percentile phantom section wrapped in plastic and centered in a mold.

Once all 18 molds were complete, the 50th percentile phantom section associated with each was wrapped tightly in plastic wrap to protect it from the liquid ATES material. Each phantom section was then appropriately centered in the mold, using bones and the segmented 90th percentile phantom dataset as landmarks. A centered slice in a mold is shown in Fig. 4.

**Figure 5:**
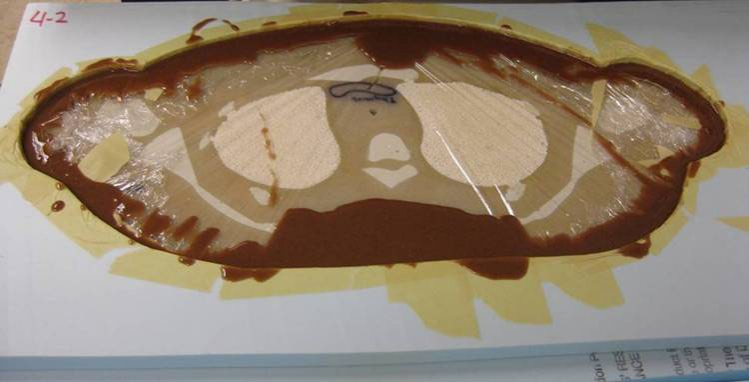
90th percentile phantom mold filled with ATES material.

The adipose tissue‐equivalent substitute material was prepared, using an electric drill with a mixing bit to ensure adequate mixing of the phenolic microspheres in the urethane material. The ATES material was then poured into the molds around the existing 50th percentile phantom sections and allowed to cure. No release agent was needed for the molds prior to pouring the ATES material. A photograph of this step of the process is shown in Fig. 5.

**Figure 6:**
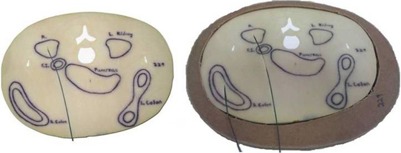
Cross‐sectional slice of the original 50th percentile phantom (left) and the completed 90th percentile phantom addition (right) at the same phantom section. Fiber‐optic coupled dosimeters are shown positioned in various organ locations.

**Figure 7:**
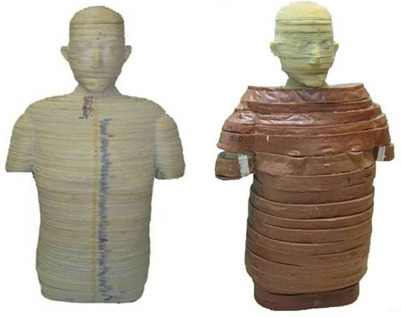
Original 50th percentile anthropomorphic phantom shown alongside the same phantom with an ATES addition, producing a 90th percentile by weight phantom. Note the reduction in z‐axis resolution in the addition as compared to the original phantom.

After allowing the ATES material to cure overnight, the phantoms were removed from their molds. The 90th percentile addition sections were mechanically cleaned of any excess material. The plastic wrap was removed from the 50th percentile phantom sections and any ATES that may have leaked though was also removed. A completed section of the 90th percentile phantom is shown alongside the same section of the original 50th percentile phantom in Fig. 6, and the completed 90th percentile phantom is shown alongside the original 50th percentile phantom in Fig. 7.

In subsequent CT imaging of the completed 90th percentile phantom, the ATES material was found to have an average HU value of ‐100 throughout the length of the phantom. No evidence of settling was seen in the completed phantom sections, and HU values were uniform both throughout a single phantom section and from section to section. The PMC 121/30 Dry base material is sufficiently viscous and cures fast enough that settling of the microspheres has not been a problem in phantom construction, provided there is adequate mixing of the materials.

## III. RESULTS & DISCUSSION

A commercially available urethane rubber product was mixed with low‐density phenolic microspheres in order to produce an adipose tissue‐equivalent substitute material with X‐ray attenuation properties and density simulating that of subcutaneous fat. This ATES material is initially a viscous liquid capable of being poured into molds, but cures in a matter of hours to a rubber that has proven to be robust and durable over an extended period of time. The cured material holds its shape well and can easily be transported without worry of tearing or breakage. It is also flexible and easily cut, allowing for placement of various dosimeters within the phantom without the creation of large air gaps.

The specific recipe for ATES material in this work produced a material that matched the HU value for a 120 kVp CT beam. For applications where the X‐ray beam quality is significantly different, it is likely that modifications to the mixture would be needed, though the same methodology could be used to find an appropriate ratio of materials. It is possible that for beam qualities drastically different than that used in this study, an entirely different base material or additive would be needed.

The process described in this work utilized a custom‐built anthropomorphic phantom as the base, and hybrid‐computational datasets and an engraver for creating the outer body contours of the addition. However, the same general methodology could be used with any of the commercially available anthropomorphic phantoms, or even with standard CTDI phantoms. Furthermore, the molds for adipose additions could be created without the engraver or computational datasets. The foam insulation board, which is inexpensive and readily available at most hardware stores, is easily cut with a razorblade, so outer body contours of the molds could be cut by hand. It would take an estimated one to two days to hand‐cut the molds with a razorblade for a full sized adult phantom. The number of ATES addition sections created for a given phantom can be altered, depending on the particular application, keeping in mind the trade‐off between z‐axis resolution of the completed phantom and the time and effort spent during fabrication. Excluding the costs of the engraving system (approximately $12,000), the total construction costs for the ATES addition was approximately $700.

## IV. CONCLUSIONS

A unique methodology has been developed to construct adipose tissue‐equivalent additions for anthropomorphic phantoms. These additions allow institutions to inexpensively expand their use of existing phantoms for radiation dose measurements by better modeling patients of various body mass indexes. In this case, an addition to make a phantom representing a patient with a BMI of 32.5 was created from an existing phantom with a BMI of 26.1. Potential uses for phantom additions of various sizes could include investigating the effects of patient size on organ doses and image quality during tube current‐modulated CT scans, information that could aid in the optimization of patient size‐specific CT protocols across various scanners.
